# Voronoi diagrams and Delaunay triangulation for modelling animal territorial behaviour

**DOI:** 10.1002/ece3.11715

**Published:** 2024-07-23

**Authors:** Rhainer Guillermo‐Ferreira, Alexander E. Filippov, Alexander Kovalev, Stanislav N. Gorb

**Affiliations:** ^1^ Department of Biological Sciences Federal University of Triangulo Mineiro Uberaba Minas Gerais Brazil; ^2^ Donetsk Institute for Physics and Engineering National Academy of Sciences of Ukraine Donetsk Ukraine; ^3^ Functional Morphology and Biomechanics, Zoological Institute Kiel University Kiel Germany

**Keywords:** agent‐based model, agonistic, contest, ecology, evolution, tessellation

## Abstract

We explore the use of movable automata in numerical modelling of male competition for territory. We used territorial dragonflies as our biological inspiration for the model, assuming two types of competing males: (a) faster and larger males that adopt a face‐off strategy and repulse other males; (b) slower and smaller males that adopt a non‐aggressive strategy. The faster and larger males have higher noise intensity, leading to faster motion and longer conservation of motion direction. The velocity distributions resemble the Maxwell distributions of velocity, expected in Brownian dynamics, with two probable velocities and distribution widths for the two animal subpopulations. The fast animals' trajectories move between visually fixed density folds of the slower animal subpopulation. A correlation is found between individual velocity and individual area distribution, with smaller animals concentrated in a region of small velocities and areas. Attraction between animals results in a modification of the system behaviour, with larger animals spending more time being surrounded by smaller animals and being slowed down by their interaction with the surroundings. Overall, the study provides insights into the dynamics of animal competition for territory and the impact of attraction between animals.

## Graphical abstract

Modelling competition for territory in animals is widespread technique but lack the implementation of multiple variables in editable scenarios. We used movable automata, inspired by territorial dragonflies to provide new insights into the dynamics of animal competition for territory and the impact of attraction/repulsion between animals.

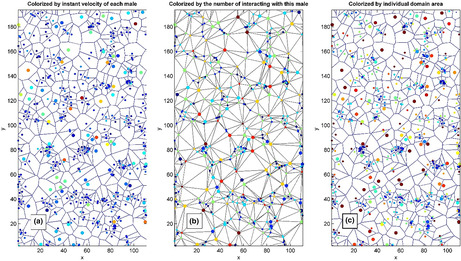

## 1 INTRODUCTION

Territorial behaviour is widespread among animals, particularly males. Territories offer advantages such as access to resources and females and create asymmetries between males with different qualities and strategies. Understanding the dynamics of such complex structures requires considering variations in animal behaviour and interactions between rival males. In this scenario, multiple male strategies may interact and counteract turning the study of the dynamics of territory ownership and home‐range sizes into a hard—and perhaps impossible—task in the field, hence, a great deal of research has been done on modelling animal territorial behaviour (Davies & Houston, 1984). Modelling animal behaviour using automata‐agent‐based models provides a more accurate representation of animal behaviour and can predict the occurrence and evolution of territoriality (An et al., 2021). However, these models do not consider spatial relations or individual mobility. New models that incorporate these factors can increase model precision and be validated through field or laboratory experiments.

Here, we developed a model applying the Voronoi diagram and Delaunay triangulation to address questions regarding animal territoriality. The model utilises the nearest‐neighbours concept to predict attribute values based on spatial interactions of neighbour agents forming Voronoi polygons. Each sample, a territorial male in our case, is positioned at the centroid of a polygon (a territory in our model), such that each male is nearer to the borders of its territory than to other males (Aurenhammer et al., 2013; Boots et al., 2009; Legendre & Legendre, 1998). Such spatial interpolation models have been previously used in life sciences for modelling forest spatial dynamics (Galitsky, 1990) animal collective behaviour (i.e., swarming, schooling, herds; Klamser & Romanczuk, 2021; Kolpas et al., 2013; Rahmani et al., 2020), animal movement and home‐range estimation (Casaer et al., 1999; Downs & Horner, 2009), and animal population dynamics and habitat occupancy (Giavazzi & Vailati, 2014; Stewart & van der Ree, 2010; Wakefield et al., 2013; Wilkin et al., 2006).

Modelling animal territoriality using Thiessen or Voronoi polygons dates to the studies by Hasegawa and Tanemura (1976, 1980) and Adams (1998, 2001), when geometrical models were built to estimate individual distribution and territory shape and size of territorial animals (reviewed by Schlicht et al., 2014)—not much dissimilar from studies on population dynamics and home‐range estimation. Recently, Voronoi diagrams have also been used in field studies on animal territoriality and aggression (Collis, 2022). Nevertheless, although this kind of modelling has been used previously to study animal territoriality, numerous possible behavioural strategies may result in the spatial dynamics of male territories, have different biological meanings, and impose different costs.

Therefore, here we propose using Voronoi diagrams and Delaunay triangulation to study the spatial resolution of agonistic interactions in territorial animals. We applied automata‐agent‐based models with movement to consider different male strategies of territory patrolling and defence. Although our model can be used to study the territorial behaviour of any given taxa, to better illustrate and contextualise its use, we give an example of the possibilities of our modelling using the case of dragonflies and damselflies (Odonata), which are common biological models in studies of animal territoriality.

### 1.1 Biological background and inspiration for the model

It is quite common to find male odonates engaging in non‐contact aerial displays. In most cases, such encounters boil down to face‐offs, when both males face each other in an aerial display imitating each other's movement before one withdraws (Sirot et al., 2003). These encounters are usually brief and end in less than 1 min, often less than 10 s. Some may argue that this behaviour is used by males to avoid male–male sexual interactions, working as a sexual recognition visual signal (see a similar argument for male colouration, Beatty et al., 2015; Khan & Herberstein, 2019). This would be the case in most non‐territorial damselflies, especially in species where males lack the ability to correctly recognise mates. Nevertheless, males of some odonate species are site attached or even territorial. In this case, face‐offs and other male displays may function as ward‐off or aggressive displays that repel intruders. Hence, males may guarantee site or territory ownership with such displays and, consequently, create a hierarchy among males of the same species across suitable oviposition sites that ultimately dictate access to females.

In recent research, Pinto and Peixoto (2019) classified agonistic interactions among animals into non‐aggressive, quasi‐agonistic, and true agonistic encounters. Male–male interactions in odonates, including the face‐off display, can be categorised as non‐aggressive if they serve as intrasexual recognition signals only. Male–male encounters can also be quasi‐agonistic if the intruder males are probing the territory or resource. True agonistic encounters involve an assessment of rival fighting abilities and typically escalate into long contests. Brief encounters between male odonates may not be agonistic because males cannot impose clear costs on their rival. However, costs related to territory ownership and access to females must be considered. This study examines whether male–male interactions in odonates can be considered “truly” agonistic if we consider spatial distribution and territory size as the implied costs and benefits of conflict resolution.

For this, we built a simple model considering two male strategies: (i) percher; and (ii) flier. These are two common strategies in odonates that can variate among species (de Almeida et al., 2022) and even among males of the same species (Vilela, Del‐Claro, & Guillermo‐Ferreira, 2017; Vilela, Tosta, et al., 2017). Percher males usually exhibit low mobility and perch on the vegetation at streams and ponds and wait for females to arrive, while flier males are more active and patrol the territories searching for females. Flier and more active males tend to be larger (see a real example in Vilela, Del‐Claro, & Guillermo‐Ferreira, 2017; Vilela, Tosta, et al., 2017), hence, we considered so in our model. In the second step of our model, we included longer male–male interactions provoked by larger and more agile males (fliers) that spend more time repelling rivals. For a real example of damselflies engaging in longer fights according to male strategy, see Guillermo‐Ferreira et al. (2015) and Pestana et al. (2018).

In this scenario, we make a distinction between short‐duration face‐offs (our first model) and long‐duration aerial contests (our second model). Males of *Argia reclusa* are very responsive to the presence of other males, and always dash towards intruders and try to repel rivals (Guillermo‐Ferreira & Del‐Claro, 2012). These damselflies never engage in long contests, like *Hetaerina* (Guillermo‐Ferreira & Del‐Claro, 2011a), *Chalcopteryx* (Guillermo‐Ferreira et al., 2014) or *Mnesarete* (Guillermo‐Ferreira et al., 2015). In this species—and many other site‐attached/territorial ones—males touch the rival for a small instance or engage in face‐off displays (Guillermo‐Ferreira & Del‐Claro, 2012). The same can be observed in *A. tinctipennis* (pers. obs.), some *Oxyagrion* (Guillermo‐Ferreira & Del‐Claro, 2011b) and *Tigriagrion aurantinigrum* (Vilela, Del‐Claro, & Guillermo‐Ferreira, 2017; Vilela, Tosta, et al., 2017), for example. Male–male interactions result in the establishment of a hierarchy in territory ownership and female access (Guillermo‐Ferreira & Del‐Claro, 2012), since females visit the best sites to mate and oviposit (Guillermo‐Ferreira & Del‐Claro, 2011b).

In *Pyrrhosoma nymphula* (Sulzer) (Zygoptera, Coenagrionidae), territorial males drive away intruders by chasing them and, sometimes, use physical contact (Gribbin & Thompson, 1991). The same behaviour was observed in males of other coenagrionids: *Coenagrion lindeni* (Selys) (Utzeri et al., 1983), *Argia apicalis* (Bick & Bick, 1965), *A. plana* (Bick & Hornuff, 1971) and *Mortonagrion hirosei* (Watanabe & Mimura, 2004). The face‐off display can even be used by andromorphic females to mimic male behaviour and repel unwanted mates (e.g., Robertson, 1985; Van Gossum et al., 2001). In clearly non‐territorial species, the face‐off display is used by males to ward‐off males that attempt to assume tandem with them (e.g., Cordero‐Rivera, 2016) or that try to dislodge them from a tandem with a female (Guillermo‐Ferreira & Del‐Claro, 2012).

Most owner‐intruder encounters are quite different from intersexual interactions. Such encounters result in spacing between males, as winners fly back to their initial perch and losers tend to disperse. Although there is evidence that male colouration may function as aposematic signals that advertise male unprofitability as mates to other males—at least in non‐territorial species (Beatty et al., 2015; Khan & Herberstein, 2019), there is also evidence that asymmetries in male colouration may predict the outcome of male–male interactions in site‐attached species (Vilela, Del‐Claro, & Guillermo‐Ferreira, 2017; Vilela, Tosta, et al., 2017). Therefore, short‐duration contests may have evolved to avoid energetic depletion in small damselflies like coenagrionids, defining the winner with simple visual cues and simple rules for conflict resolution. Our models test whether simpler male–male encounters can be more profitable in terms of spatial distribution among males when compared to longer interactions between males.

## 2 MOVABLE AUTOMATA MODEL AND APPLICATION OF THE VORONOI DIAGRAM AND DELAUNAY TRIANGULATION TO THE NUMERICAL MODELLING OF THE MALE COMPETITION FOR A TERRITORY

A dynamic model is a way of simulating how things change over time and space. One type of dynamic model uses mobile “cellular automata” (Wolfram, 1984, 2002). This means that each thing in the model is like a moving cell that interacts with other cells. For example, we can use this type of model to study how animals move and interact with each other. Each animal has a position (in 2‐D) given by the vector ***r***_*i*_ and a momentum given by the vector ***p***_*i*_. The momentum depends on the mass of the animal. The interaction between two animals depends on how far apart they are, which is given by | ***r***_*i*_‐***r***_*j*_|. The interaction also has a potential energy *U*(***r***_*i*_‐***r***_*j*_). We can write all this in a mathematical formula called the Hamiltonian:

(1) $$H\left(r_{i},p_{i}\right)=\sum_{i=1}^{N}p_{i}^{2}/2m_{i}+\sum_{i,j=1}^{N}U\left(\left|r_{i}-r_{j}\right|\right)/2.$$

When the animals are not moving, they have some positions that balance the forces that push them away or pull them together. These forces depend on how close or far the animals are from each other. We can use a mathematical function called the Gauss potential to describe these forces. The Gauss potential has two parts: one for repulsion and one for attraction:

(2) $$U\left(\left|r_{i}-r_{j}\right|\right)=C_{\mathrm{ij}}\;\exp\left\{-{\left[\left(r_{i}-r_{j}\right)/c_{\mathrm{ij}}\right]}^{2}\right\}-D_{\mathrm{ij}}\;\exp\left\{-{\left[\left(r_{i}-r_{j}\right)/d_{\mathrm{ij}}\right]}^{2}\right\}.$$

The Gauss potential has some parameters that control how strong and how wide the forces are. Cij and Dij are the strengths of attraction and repulsion, respectively. cij and dij are the distances at which the forces are strongest. The animals want to be at a distance that makes the energy as low as possible. This means that Cij should be much bigger than Dij, and cij should be smaller than dij.

At first, we do not know where the animals will end up. We must let them move around until they find their best positions. We can do this by putting them randomly on a rectangle with sides [0, Lx] and [0, Ly]. If nothing else makes them move, they will eventually stop moving and form a stable pattern. The pattern depends on how we treat the edges of the rectangle. Sometimes we want to pretend that the rectangle is part of a bigger system that goes on forever. In that case, we can use periodic boundary conditions. This means that when an animal goes out of one side of the rectangle, it comes back in from the opposite side. The same thing happens for the forces between animals: we use the shortest distance between them or their copies on the other side of the rectangle.

One way to model this problem is to imagine that the system is trapped inside a box with walls that bounce back any particles that hit them. These walls are located at y = 0 and y = Ly for the horizontal direction, and x = 0 and x = Lx for the vertical direction. The bouncing effect of the walls can be described by functions that depend on how far away the particles are from them: Uup = C·exp[(y‐Ly)/c] and Udown = C·exp[−y/c] for the horizontal walls; Uright = C·exp[(x‐Lx)/c] and Uleft = C·exp[−x/c] for the vertical walls. These functions are called reflecting boundary conditions1. The larger C and smaller c are, the more rigid and sharp are the walls compared to other forces and lengths in the system.

Of course, there is no such box with perfect walls. The system might be limited by some natural features like a hill, a river, or a forest. In that case, we need to use different boundary conditions that match those features better. But here we use this simple box model as an approximation.

The system can have any number of components (types of particles) if we specify their parameters Cjk and cjk. These parameters determine how big and strong each component is, and how much they repel (or attract) each other. To make our model more general and realistic, we can choose different values of Cjk and cjk within some range based on real data about different types of particles.

However, to make this preliminary study as simple and transparent as possible and concentrate on its main goals, we will take only two kinds of males in the population. It means that only the combinations of *C*_*jk*_, *c*_*jk*_, like *C*_*11*_, *C*_*12*_, *C*_*22*_ and *c*_*11*_, *c*_*12*_, *c*_*22*_ exist here. To get reliable results and test how well our model works, we changed the starting locations of the two types of animals every time we ran a new simulation.

The simplicity of the equations of motion

(3) $$m_{i}\partial v_{i}/\partial t=-\partial H\left(x_{i},p_{i}\right)/\partial p_{i}=f_{i},$$

is deceptive. Using equations that involve all possible interactions between animals can be very slow and inefficient, especially if we must consider periodic boundary conditions that create imaginary copies of animals. But if we assume that animals only interact with those they can see within a certain distance (or within their personal territories), we can simplify the problem a lot. We can do this by using a Voronoi diagram, which is a way of dividing the space into regions based on how close each point is to an animal. Each region belongs to one animal and contains all the points that are closer to that animal than any other. These regions are called Voronoi cells. This means that each animal only interacts with its nearest neighbours, which are those animals whose Voronoi cells share a border with its own cell. Dragonflies and damselflies, although exhibit incredible visual capacities, usually focus on direct neighbours and intruders during territorial contests.

The Voronoi diagram approximates our dynamical model, with each cell having proximate neighbours determined by the Delaunay triangulation. This triangulation ensures that no point in the set is within the circumcircle of any triangle, providing a realistic representation of motile automata (animals) and their neighbours. The result is a concise list of neighbours interacting with each male. Visual impressions of the structures of the Voronoi and Delaunay diagrams are depicted in the figures and movies below. In general, interacting entities exchange their momentum of motion in their collisions, and thus a dissipation channel acting to equilibrate their relative velocities (that occur to be within the distance $c_{v}$ close to the equilibrium one) needs to be introduced. This can be done by incorporating an additive velocity‐dependent force

(4) $$f_{i}^{v}=\eta \sum_{j=1}^{N_{\text{neigbours}}}\left(v_{i}-v_{j}\right)\exp\left[-{\left[\left(r_{i}-r_{j}\right)/c_{v}\right]}^{2}\right],$$

influencing the *i*‐th particle, with some dissipation coefficient *η*. Consequently, the equations of motion acquire the following configuration:

(5) $$m_{i}\frac{\partial v_{i}}{\partial t}=f_{i}^{r}-f_{i}^{v}.$$

In numerical computations, one should employ Verlet's method to integrate them, which preserves the energy of the system at each time step in the absence of dissipation and external energy sources and ensures the stability of the numerical computations below.

To simulate a source of motion in our model, a random source of noise of an unspecified nature is used, like the one which is normally applied in the modelling of the Brownian dynamics. It is the so‐called Langevin noise. In general, if a temperature *T* of some thermal bath is non‐zero, an array of random δ‐correlated Langevin forces is included in the equations of motion. According to the fluctuation‐dissipation theorem it is defined as follows:

(6) $$\left\langle \xi \left(t,x_{i},y_{i}\right)\xi \left(t^{\prime },x_{j},y_{j}\right)\right\rangle =\mathrm{D\delta}\left(t-t^{\prime }\right)\delta_{\mathrm{ij}}$$

(7) $$\left\langle \xi \left(t,x_{i},y_{i}\right)\right\rangle =0,$$

where $D=2\gamma k_{B}\mathrm{Tm}/\mathrm{dt}$, $k_{B}$ is the Boltzmann constant, γ is a dissipation constant, and dt is the discrete time step.

**FIGURE 1:**
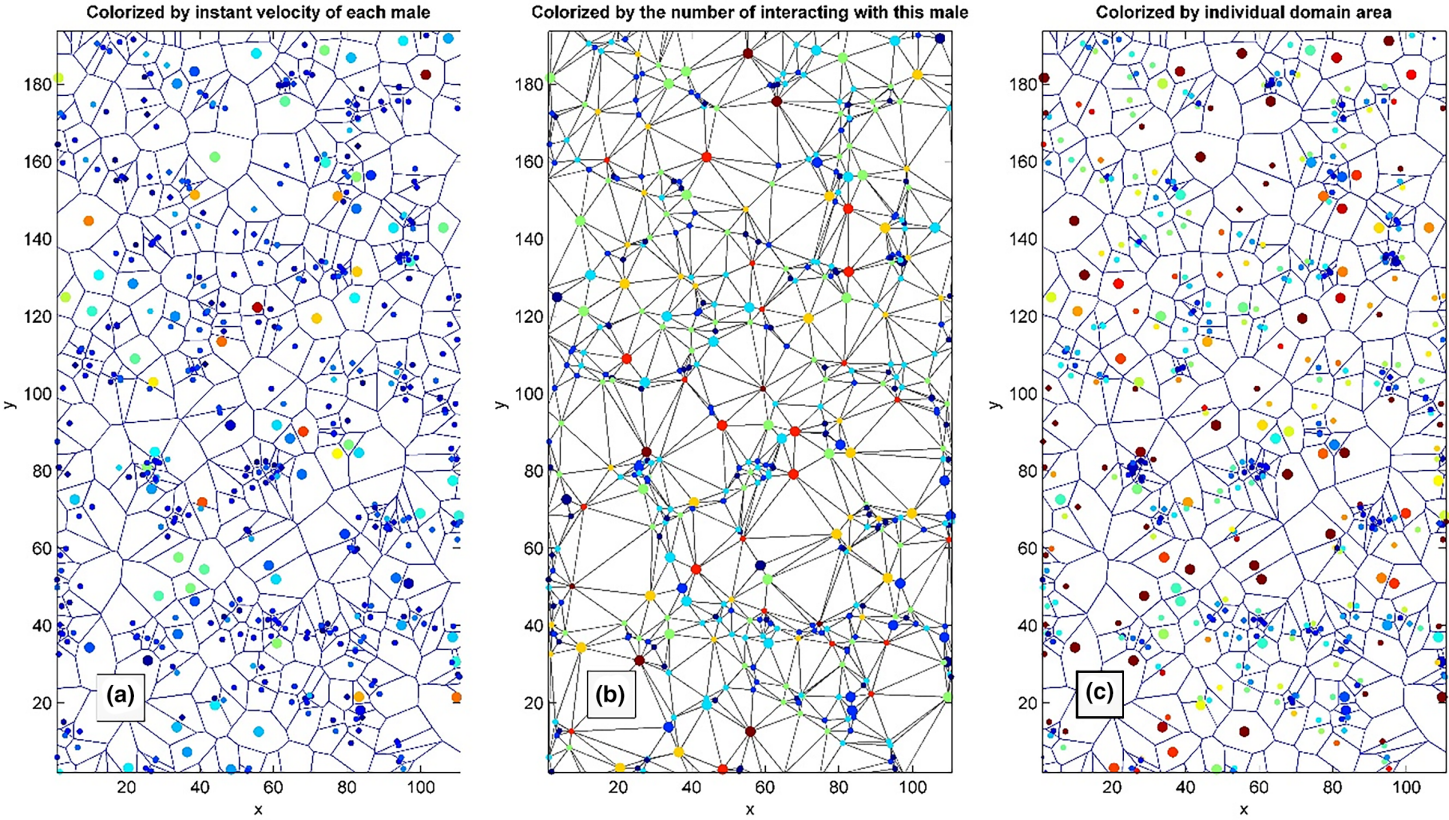
Typical instant configuration of the system with the movable automata coloured differently according to their velocities (a), number of interacting neighbours (b) and areas of individual domains (c), respectively. The larger and faster (flier) males repulse other males and are shown as larger circles, smaller circles represent a slower/stationary strategy (percher males). The standard MatLab colour map is used for the visualisation (the larger values are shown by the red and the smaller ones by the blue colours, respectively). The interaction parameters applied for this picture are chosen as follows: $C_{1}=1$, $c_{1}=1$, $C_{2}=2$, $c_{2}=4$. To get different mobilities, different noise intensities and masses were used for different kinds of animals: ${\mathrm{kT}}_{1}=1$, ${\mathrm{kT}}_{2}=10m_{1}=1$ $m_{2}=4$.

Independently on the presence of thermal source, the interaction between the animals (movable automata) modifies their kinetic energy and leads to both effects: additional thermalisation of the system, because of its non‐linearity and its self‐organisation. It motivates us to an application of the following visualisation technique which we will exploit below. Specifically, we employ plotting of the kinetic energy of the “particles” by the artificial colours. In this approach, one obtains a kind of motile colour map on a non‐uniform grid in the representation that is natural for the discrete model based on the motile automata. The results attained in the framework of this model are shown in Figure 1. This was done to represent the different strategies adopted by the biological model.

As we have already stated above, it is natural for this problem to construct a Voronoi diagram where each cell corresponds to a domain occupied by one animal that interacts with other animals identified using the associated Delaunay triangulation. From the mathematical point of view, each cell is a polygon with a quantitatively defined area. This area can be certainly calculated at every time step. The number of the neighbours found according to the Delaunay procedure can be also directly counted for every cell (and respectively, for every animal). Both these features give rather important information about an instant configuration of the system and should be visualised as well. Counting that each of these three values is associated with every automaton, one can plot all of them as a colour of the movable points according to the corresponding method.

Typical instant configuration of the system with the movable automata coloured according to their velocities, number of interacting neighbours and areas of individual domains is shown in the subplots (a)‐(c) of Figure 1. The fast animals are shown as large circles. This is one more characteristic (which might correlate with velocity and territorial behaviour) of every individual. For simplicity, this characteristic is artificially restricted to just two types in this paper. In the general case, the sizes of the animals are continuously distributed from maximal to minimal ones. Thus, a spectrum of the circle sizes should be used for such a general system. This spectrum, in fact, has the same meaning as the colour spectrum of the in MatLab colour map and in principle, the sizes can be coloured too. However, it seems rather natural, to display the size of the individuals by the size of the symbols.

The standard MatLab colour map is used for the visualisation (the larger values are shown by the red colour and the smaller ones by the blue colour, respectively). The interaction parameters for simulation screens shown in Figure 1 were chosen as follows: $C_{1}=1$, $c_{1}=1$, $C_{2}=2$, $c_{2}=4$. To get different mobilities, the different noise intensities and masses are used for different kinds of animals: ${\mathrm{kT}}_{1}=1$, ${\mathrm{kT}}_{2}=10$, $m_{1}=1$, $m_{2}=4$. The same parameters are applied in all the cases below if something is not additionally specified.

**FIGURE 2:**
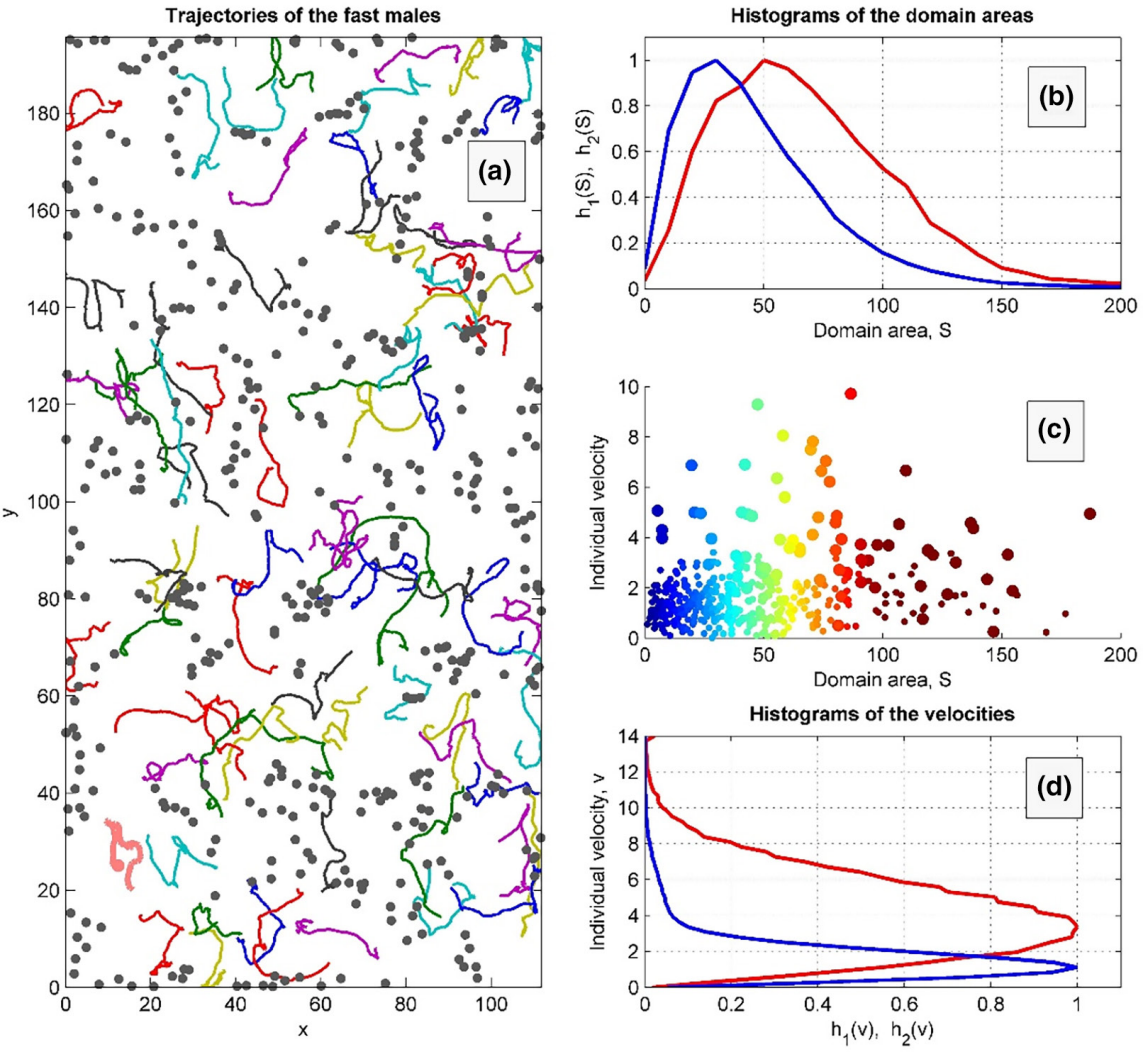
Instant positions of perchers (grey points) and the trajectories of flier males are shown in the subplot (a) by so‐called ‘comet‐tails’. Time‐accumulated distributions of the individual domain areas and velocities of fast and slow subpopulations are plotted in the subplots (b) and (d) by red and blue curves, respectively. Instant scatter plot in the plane area‐velocity consisting of the large and small points colourised according to the distribution of the domains is presented in subplot (c). “h” = normalised probability for the groups “1” and “2”, correspondingly; “S” = area, and “v” = velocity.

The higher noise intensity of the faster fliers accompanied by their higher body mass leads to the desirable effect of faster motion together with a longer time‐conserving motion direction. It was expected because the higher mass corresponds to the stronger inertia under the influence of both factors: the random noise and mutual collisions between the animals of different masses. This supposition was checked by us in the frame of the model numerically, as well. Generally, one can observe (see subplot (d) in Figure 2) that it leads to the velocity distributions looking like the Maxwell distributions of the velocity, which is expected in Brownian dynamics with two different mostly probable velocities and distribution widths for the two animal subpopulations.

The Figure 2 also illustrates the difference in motion of the faster/larger males and smaller/slower ones. It is illustrated in subplot (a), where we plot short parts of the individual trajectories (so‐called “comet‐tales”) of the fast movable automata, which are moving between almost visually fixed density folds of the subpopulation of slower animals. The same difference in motion is even clearer in Movie S1, which reproduces in dynamics all the aspects of the process simultaneously. This movie also shows how time‐dependent distributions of the velocities and individual domain areas are accumulated with time. Stationary distributions obtained in a stationary process limit are shown in the subplots (b) and (d) of Figure 2.

In the context of the present paper the most important observation is the correlation between characteristic individual velocity and individual area distribution, which is illustrated in the subplot (c) of Figure 2. One can see that at every time moment the small circles (describing slower and smaller animals) are mainly concentrated in a region of the small velocities and areas, while the larger ones outline them in outer belt of this phase portrait. For convenience, the circles are additionally colourised by the colour map corresponding to the sizes of individual areas.

An alternative description of personal domain area in the framework of the Delaunay‐Voronoi approach is to calculate the occupation density for both populations. To avoid any misunderstanding, it is important to note that this calculation is based on the same Delaunay definition of the neighbours, interaction with which is considered in the equations of motion. From the instant distribution of animals their spatial density may be formally evaluated using standard Gaussian convolution (as it is normally used in many Brownian dynamic or population dynamic studies).

**FIGURE 3:**
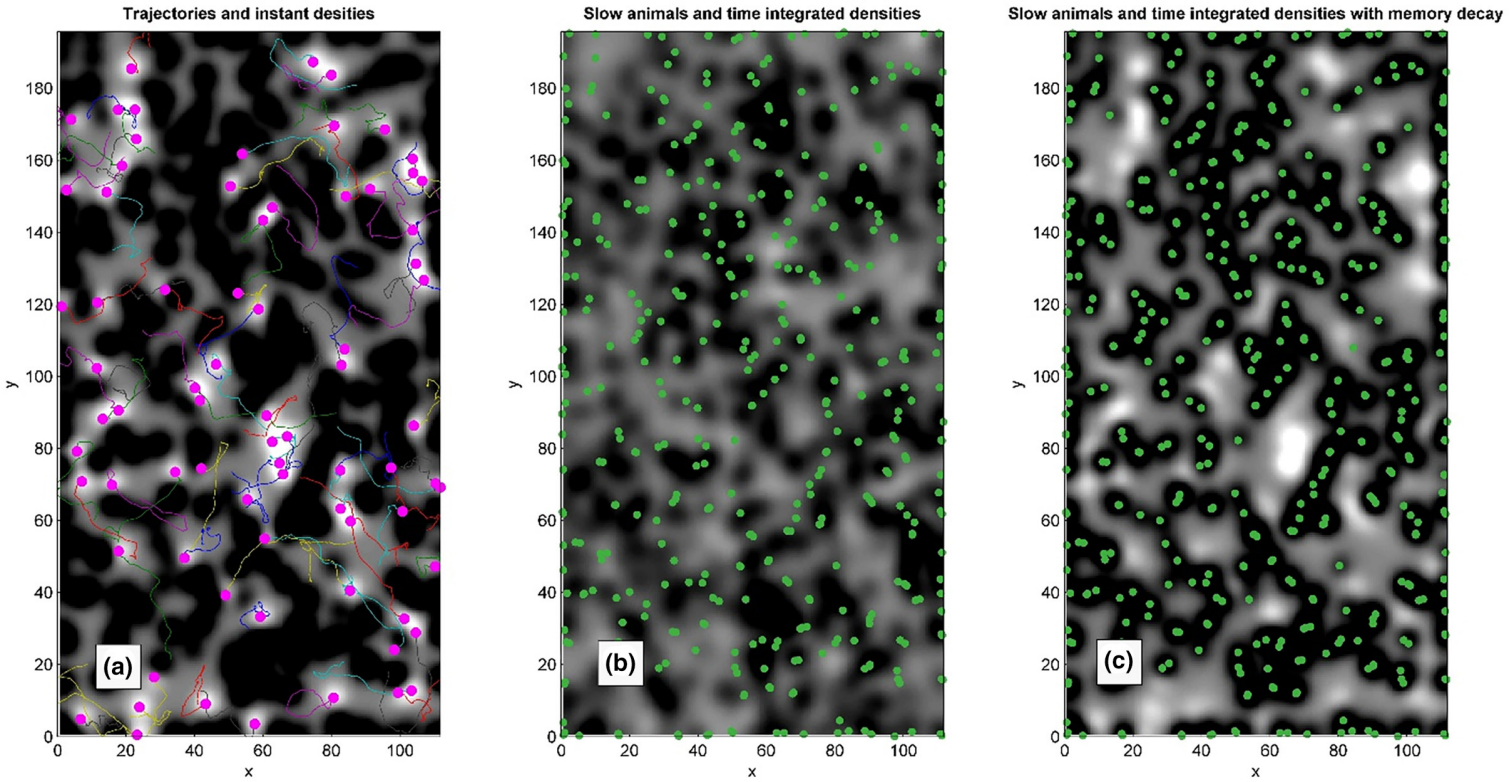
Distribution of the difference between the density of space occupied by the fast and slow males shown by greyscale, where large values correspond to the white colour. Instant positions of the fast and slow subpopulations are shown by the magenta and green points of different sizes, respectively. Subplot (a) reproduces instant occupation density distribution. Subplot (b) presents the distribution integrated over complete run of the process. Subplot (c) shows the result of the integration with memory decay, respectively.

Such a procedure is formally applied by us to get the density distributions presented in Figure 3. An evolution of these densities in the dynamics is reproduced in Movie S2. A mutual correlation between the positions of the animals and densities is clearly seen in the static figures and movie. To make the figures more readable, we used greyscale colour map to represent the density maps and the large magenta/small green points to represent the strong and the weak animals, correspondingly. Instead of presentation of densities for both strong and the weak animals we present the densities difference. As a result, bright regions correspond to the territories occupied by the strong animals and dark regions correspond to the weak ones, respectively.

One can integrate the densities over time. It is expected that each integrated density will become smoother (with smaller contrast in the plot). In the case of the faster motion of individuals, a stronger smoothing is expected. In contrast, slower/percher individuals do not leave the regions occupied by them for much longer time periods. As result, even being integrated over long run time, the density conserves some memory about its initial or previous spatial distribution. In any case, both subsystems are moving, and memory about the initial (randomly chosen) configuration must disappear with time. Theoretically it allows to calculate effective per capita area for the subpopulations. However, this approach is useless for biological systems, since the necessary for that observation time normally exceeds the lifetime of local populations. Therefore, it is reasonable to calculate time averages with a memory decay. The approaches using different integration time are illustrated in subplots of Figure 3.

The proper choice of the integration time is especially crucial when the densities produced by the fast subpopulation have much smaller number of animals: 15% of the faster ones and 85% of the slower ones in the model realisation. Being formally integrated over the whole simulation time, the density of fast animals becomes completely smoothened in the map presenting the densities' difference, Figure 3. Moreover, even a slowly moving subsystem gradually leaves its initial configuration. As a result, the dark areas in time‐integrated density slowly deviate from the actual distribution. In contrast, a time‐averaging procedure performed with memory decay smoothens the distributions (minimising the fluctuation of areas per capita) and conserves the mutual correlation between the integrated densities' difference and actual distributions of the individuals. In Movie S2, one can observe such a correlation in dynamics.

**FIGURE 4:**
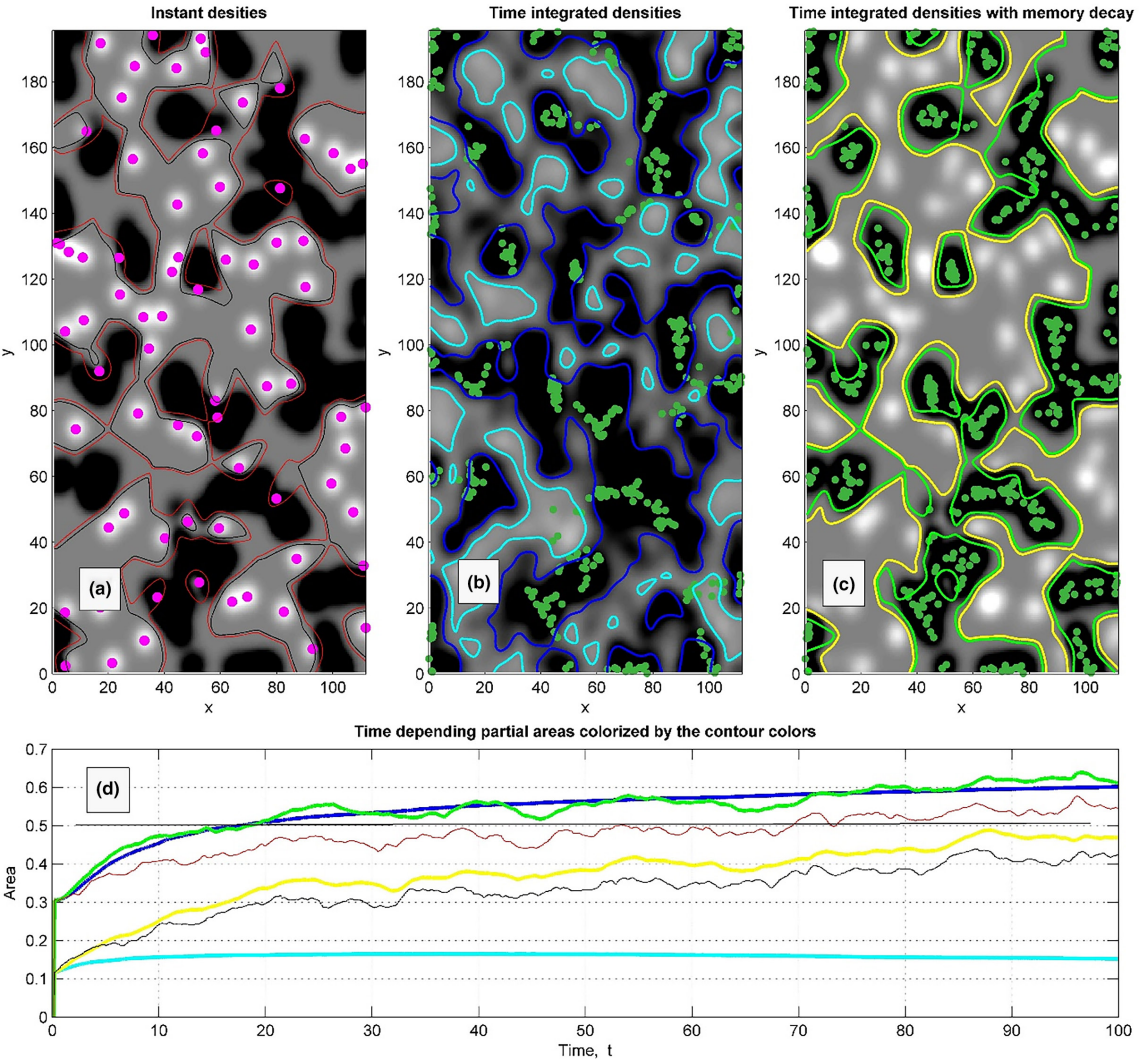
The same densities as in Figure 3 are accompanied by two kinds of contour plots which separate the areas, where densities of the fast and slow populations are equal (their difference is equal to zero), These contours are shown by black, cyan and yellow colours in the subplots (a), (b) and (c), respectively. Counting that number of the individuals in the populations are different (*N*_1_ = 15% of fast and *N*_2_ = 85% of slow in the model realisation), to calculate total weighted densities, they are renormalised in proportion *N*_1_/*N*_2_. Corresponding contours of the effective areas are shown by the brown (possibly red), blue, and green colours, respectively. Time‐dependent total areas per capita, averaged starting from the beginning of a run, are plotted in the final subplot (d) by the curves of the same colours as the contours above. Equilibrium corresponding to equivalent territories per capita is marked by the horizontal black straight line in the same subplot (d).

The same densities as in Figure 3, accompanied by two kinds of the contour plots which separate the areas, where densities of the fast and slow populations are equal (their difference is equal to zero), are plotted in Figure 4. The contours corresponding to time‐averaged densities are plotted by bold lines. Time‐dependence of the total areas per capita is plotted in the subplot (d), Figure 4. The curves' colour meaning is the same as for the contour lines in Figure 4a–c.

Considering that the number of the individuals in the populations is different (*N*_1_ = 15% of fast and *N*_2_ = 85% of slow ones in the model realisation), the maximum of the difference between the time‐averaged densities of fast‐ and slow‐moving animals at long simulation time is negative. A simple way to keep the densities' difference in the areas populated by fast‐moving animals positive is to apply the density normalisation. We renormalised the total weighted densities should be *N*_1_/*N*_2_. Contours for the corresponding normalised densities are shown in Figure 4 by the black, brown, and green lines, respectively. The area inside not normalised (cyan) contours is rather small because the density in the regions occupied by the fast individuals become smoothened and small with time. However, after renormalisation, the territory occupied by the slow subsystem strongly shrinks and is reduced to the dark regions, which are surrounded by the blue bold contours.

Equilibrium equivalent territory per capita is marked by the horizontal black straight line, Figure 4d. After some transient time during which the stronger animals conquer their territories, the territory per capita (blue line, averaging without memory decay) exceeds the equilibrium value (black line). Also, the territory per capita calculated by averaging with memory decay (green line) fluctuates around the simply average density (blue line). It means that averaging with memory decay is reasonable. Also, the density map in this case much better correlates with actual animal distribution. Moreover, as it was expected, the fluctuations of the normalised instant densities (brown contours / red line) correlate with fluctuations of densities averaged with decay (green line) and even slightly exceeds the equilibrium value. So, the prediction given by the green line is reasonably correct and realistic at the same time.

**FIGURE 5:**
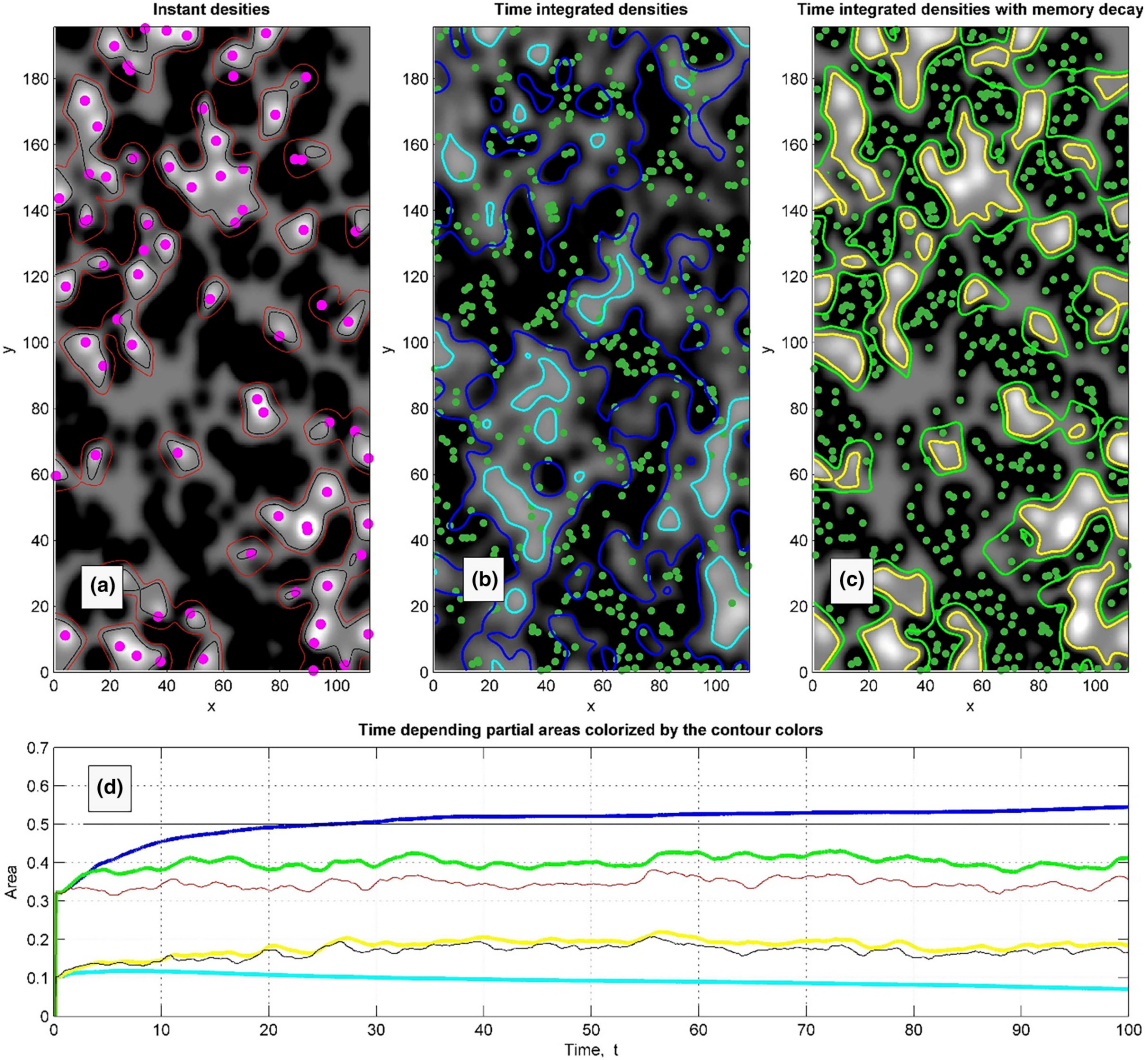
The same densities of individuals as those used in the case shown in Figure 4, but here in the case of attraction of the individuals to the (strong) aggressive ones. The colours and the meaning of the contours and curves are the same as in the previous figure.

In the model language, the non‐zero attraction means that additionally to already used positive parameter values in the interaction potential negative ones should be added: $U\left(\left|r_{i}-r_{j}\right|\right)=C_{\mathrm{ij}}\;\exp\left\{-{\left[\left(r_{i}-r_{j}\right)/c_{\mathrm{ij}}\right]}^{2}\right\}-D_{\mathrm{ij}}\;\exp\left\{-{\left[\left(r_{i}-r_{j}\right)/d_{\mathrm{ij}}\right]}^{2}\right\}$, $C_{1}=1$, $c_{1}=1$, $C_{2}=2$, $c_{2}=4$. For definiteness below, we used: $D_{1}=0$, $d_{1}=1$, $D_{2}=0.5$, $d_{2}=10$. This modification of the model immediately leads to the change of the averaged results shown in Figure 5. The most important observation here is that the averaging with memory decay (green curve) strongly deviates from the averaging over the whole history (blue curve) and descends to the equilibrium horizontal line.

**FIGURE 6:**
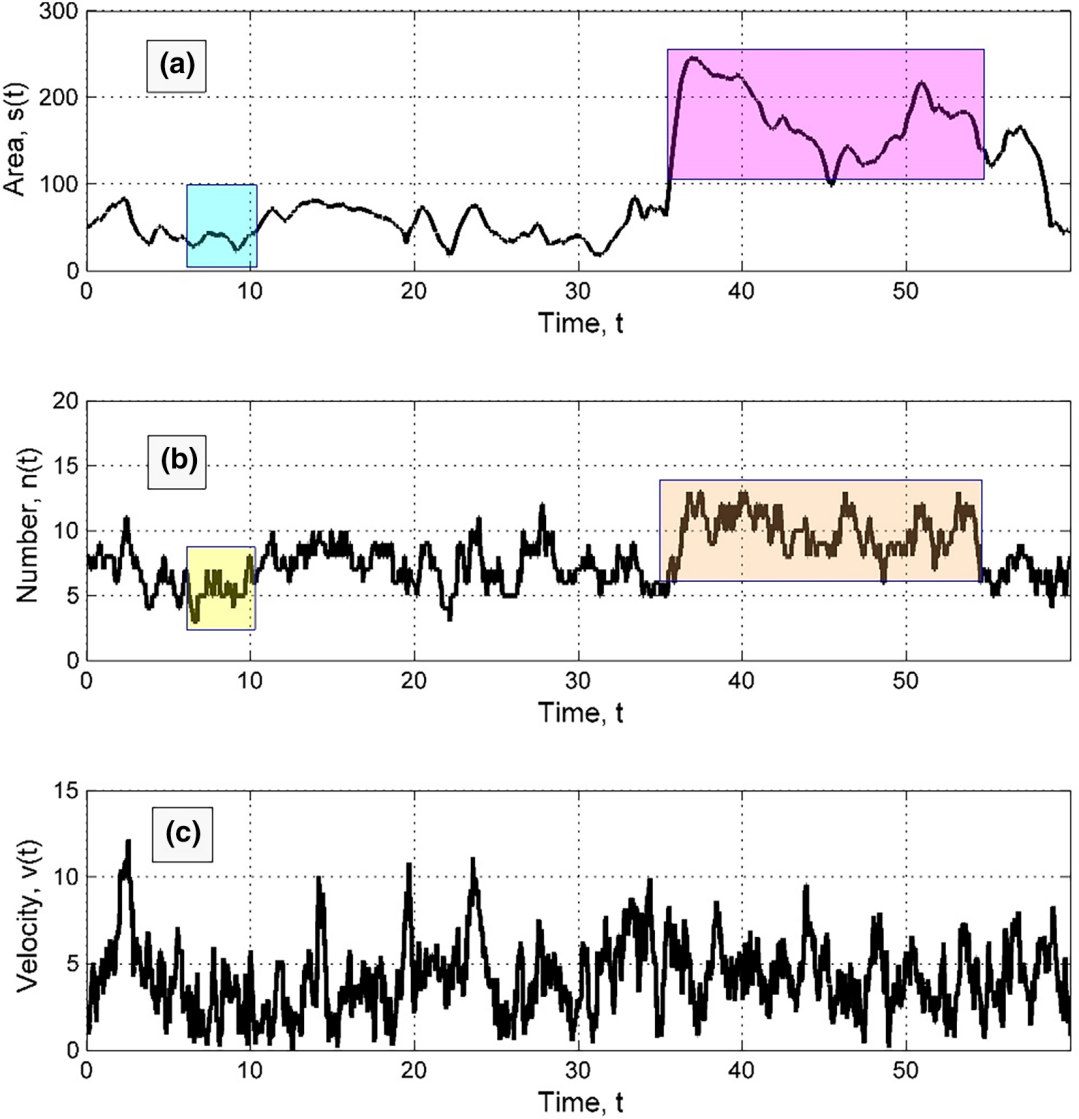
Typical time dependencies of different measures for a selected fast individual: Its personal area (a), number of the neighbours (b), and instant velocity (c). The regions of the correlations marked by the colour rectangles are described in the main text.

**FIGURE 7:**
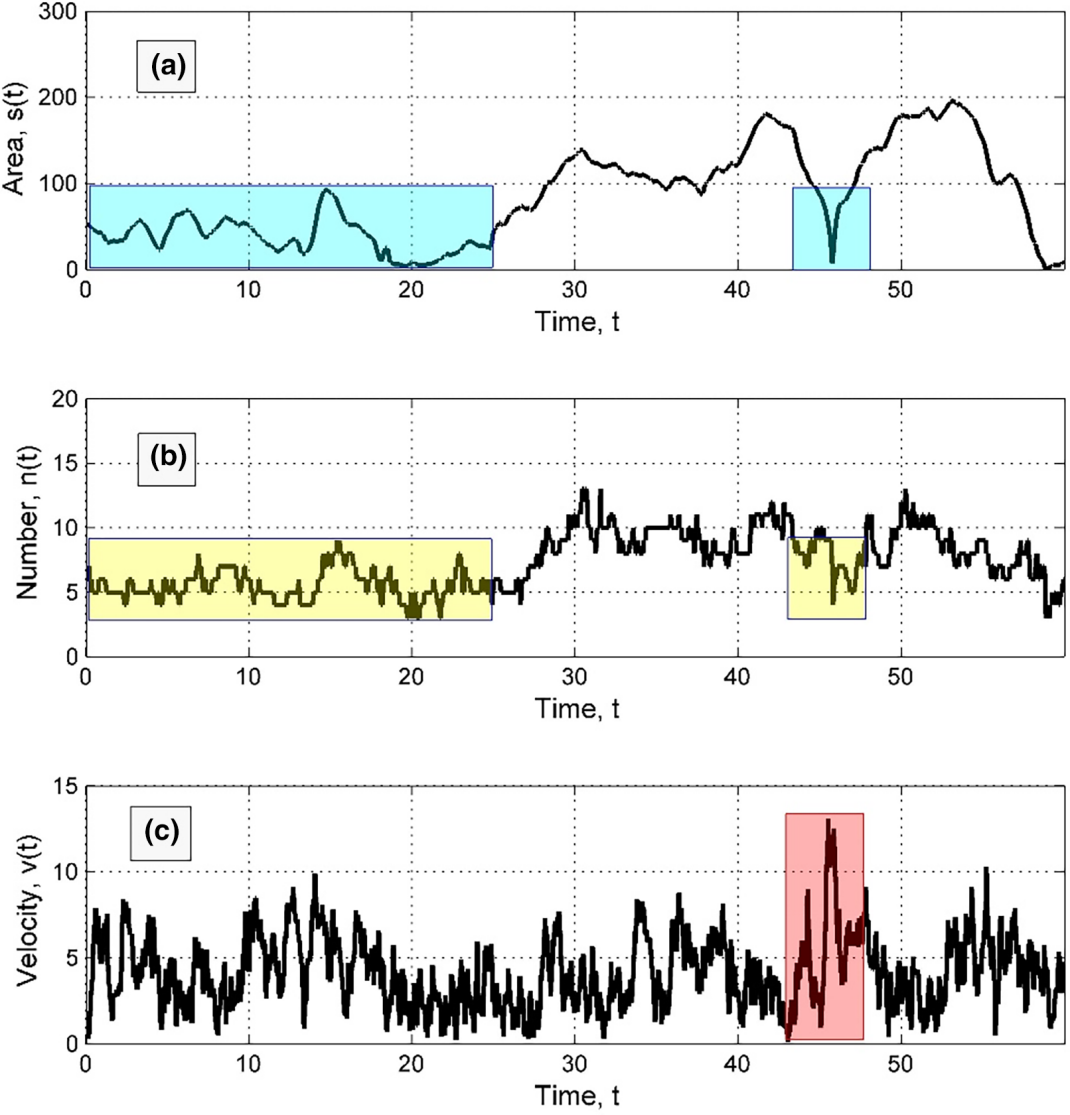
Typical time dependencies of different measures for a selected fast individual: Its personal area (a), number of the neighbours (b), and instant velocity (c) in the presence of mutual attraction. The regions of the correlations marked by the colour rectangles are described in the main text. It is important to note that the animal spends relatively long time being surrounded by the other ones. As a result, its personal area is small, and the number of neighbours fluctuates around 6 (mainly from 5 to 7). Such numbers of neighbours are typical for the hexagonal lattice with dislocations which is normal for densely packed systems.

When fliers are attracted to fight for territory, they spend more time surrounded by others, causing their average territory size to decrease. Their movement is slowed down by their neighbours, making it difficult to change their position. Movie S3 shows the changes in system behaviour. By following the motion of a chosen individual and calculating its personal area, neighbours, and velocity, the effects of attraction can be observed in Movies S4, S5 and Figures 6, 7. A smaller individual area corresponds to around 6 neighbours, typical of densely packed systems with hexagonal lattice structures. In opposite limit, when the area is very large (transparent rose rectangle), Delaunay triangulation founds a lot of neighbours *n*(*t*), mainly located on a periphery of the individual domain. This place is marked by transparent orange rectangle and *n*(*t*) fluctuates here between two times large numbers: 7 < *n*(*t*) < 14. It is also important to mention that due to strong general fluctuations of time‐depending velocities in this case, there is almost impossible to see significant correlations between *v*(*t*) and two other time‐depending values.

In the presence of mutual attraction, animals experience different dynamic processes and a static picture, as seen in Figure 7. Personal areas decrease as animals spend time surrounded by others, resulting in around 6 neighbours with blue and yellow colour rectangles. This is typical for a hexagonal lattice with dislocations in densely packed systems. However, attraction slows down interactions with dense surroundings and creates a (quasi‐)periodic environment. Animals from non‐populated areas experience dramatic drops in personal area and neighbour count when passing through walls but exhibit a surge in velocity before and after. This region is marked by a transparent red rectangle in subplot (c) of Figure 7, which is accompanied by the blue and yellow rectangles in the subplots (a) and (b) of same figure, respectively.

## 3 DISCUSSION

### 3.1 Main results

The faster fliers with higher body mass experience higher noise intensity and exhibit faster motion with longer conservation of motion direction. The velocity distribution of the animal subpopulations is like the Maxwell distribution, with two mostly probable velocities and distribution widths. Fast‐moving animals move between visually fixed density folds of slower animal subpopulations. A correlation exists between individual velocity and individual area distribution. Smaller individual domain areas correspond to approximately 6 neighbours, typical for the hexagonal lattice with dislocations in densely packed systems. Larger domain areas have fluctuating neighbour numbers of 7–14. Attraction between animals leads to modifications in behaviour, with larger animals spending more time surrounded by others and moving slower, resulting in a smaller territory than expected without attraction. Intensive moments occur when animals pass through density folds, causing a drop in individual area and neighbour count but a surge in velocity before and after passing the fold. Area averaged with memory decay strongly deviates from the area averaged over the whole simulation time and descends to the equilibrium value.

### 3.2 Voronoi diagram and Delaunay triangulation

Here we address the dynamics in conflict resolution and territorial behaviour in animals using Voronoi diagram and Delaunay triangulation. Our model can be easily powered with novel insight and features from real‐life data of male–male interactions in living species. Furthermore, multiple theoretical frameworks and backgrounds can be tested using different parameters such as male resource holding potential, resource quality, assessment abilities and strategies, female presence and quality, and environmental dynamics.

Since the first studies modelling animal territoriality (e.g., Adams, 1998, 2001; Lewis & Murray, 1993), it is known that size and shape of territories are defined by the behaviour of the agents—i.e., territorial animals or automata. Nevertheless, most models do not consider the processes behind the behavioural dynamics of animal territories (Potts & Lewis, 2014). Therefore, the kind of model we propose can be easily extended to consider and to unravel a wide range of ecological and evolutionary scenarios. Furthermore, the automata‐based model allows for increasing the complexity of game‐theoretic models, including different phenotypes to address and evaluate the consequences of interacting animals carrying the most diverse behavioural strategies—like real‐life territorial animals. In other words, territorial animals exhibit striking diverse behaviour, both genotypically and phenotypically (including phenotype plasticity). Thus, models that allow this diverse set of configurations are most needed when studying animal ecology and territoriality.

Future models must include detailed descriptions of animal movement and consequent interactions. This way, it is possible to address different questions on ecology and evolution under cost–benefit (e.g., energetics‐fitness) inputs, integrating data with cognitive maps of the environment, and even epidemics by focusing on disease spread. Generation turning may be included into the model to evaluate the frequency of alleles or behavioural strategies (or evolutionary stable strategies) generation‐per‐generation. Incorporating these modelling techniques and the immense possibilities of ideas may not only provide more accurate reasoning and evidence on animal territoriality and the dynamics before mentioned, but also the post‐hoc validation in the field or laboratory of results observed in the models.

Voronoi models have been used to test hypotheses on animal territory and home‐range area formation, however, the approach used so far is normally based on addressing which models better fit possible observations or theoretical background. The main goal of the present approach is to combine intuitive Delaunay‐Voronoi description having clear data meaning, from one side, with the rigorous fast parallel numerical simulation of the many‐body routine performed by the standard MatLab methods, differently from previous models. The kind of model we provide here seeks to understand the similarity between real‐life organisms, their behaviour, their interactions, and empirical observations. We suggest that models based on Voronoi diagrams and Delaunay triangulation is one the best choices to construct mechanistic models fill the gap needed to turn behavioural modelling in a current set of methods required for this field of science (see Potts & Lewis, 2014, for a revision of such gaps).

In principle, one can apply various approaches to the dynamic analysis of the spatially distributed population. In our previous publications, we combined the approaches differently depending on the kind of the animals. Here combination of the Voronoi diagrams and Delaunay triangulation is chosen because it seems natural for the animals that control their mutual positions and behaviour mainly visually.

An animal selects in each direction (viewing sector) limited number of the other ones, who are treated as the “closest ones”, and interacts mainly with them. Individual area which belongs to an animal is also controlled visually. Both these aspects qualitatively repeat intuitively understandable definitions of the Voronoi and Delaunay and can be simply transferred from available (already existing mathematical tools) to the study.

In the preliminary studies, we varied all the parameters many times and in wide ranges. However, the space of the parameters is multidimensional here, so for the final presentation we had to restrict ourselves only to the use of the parameters which lead to maximally realistic dynamic behaviour of the system under consideration.

### 3.3 Dragonfly model

A simple and direct application of the modelling technique presented here was shown for a real biological background. This model can be applied to any of such biological models that exhibit similar combinations of male traits while they are dispersed in an environment. In the case we used as example, male dragonflies exhibit two possible territorial strategies: (i) percher, and (ii) flier (Vilela, Del‐Claro, & Guillermo‐Ferreira, 2017; Vilela, Tosta, et al., 2017). Percher males remained clustered in certain areas, with limited dispersal and limited territorial area. The probable main fitness gain from this strategy is the probability of encountering females by adopting a sit‐and‐wait mate finding strategy (de Almeida et al., 2022) – just like web spiders catch their prey. Flier males had a higher dispersion capacity and acquired larger territories. In our model, and previous field evidence (Vilela, Del‐Claro, & Guillermo‐Ferreira, 2017; Vilela, Tosta, et al., 2017), these males are larger and can sustain longer flights and patrol a larger area, gaining access to females by increasing chance of random encounters.

By adding a second territorial trait to flier males, which is a more intolerant strategy – modelled as more time spent attracted by neighbour males – these males have apparently lost territory size in comparison to the previous mode. These results of the simplest use of the modelling technique presented here already show promising theoretical implications. In the case of aggressive males that dash towards rivals, or non‐aggressive displays such as the face‐off display in odonates, males spend more time interacting with rivals and may lose territory in such interactions.

### 3.4 Integration with empirical data

To strengthen this modelling approach, it is essential to integrate it with empirical data from field studies and experiments. Incorporating detailed behavioural data from field observations, such as movement patterns, genetic and fitness components, interaction frequencies, and territorial boundaries of specific species, can significantly enhance the model. For example, tracking individual animals using GPS or other tagging methods provides precise movement data crucial for calibrating and validating the model (Recio et al., 2011; Seidel et al., 2018). Additionally, data on population density and spatial distribution from field surveys can inform the model's initial conditions and parameters. For instance, information on dragonfly densities in various habitats can help establish realistic initial conditions for simulations (Cordero‐Rivera, 2016; Palacino‐Rodríguez et al., 2024).

Conducting controlled experiments where variables like resource availability, territory size, and the presence of competitors are manipulated (Gołąb et al., 2013) can test specific hypotheses generated by the model. Behavioural assays that measure animals' responses to different stimuli, such as the presence of rivals (Lenis & Guillermo‐Ferreira, 2020; Pena‐Firme & Guillermo‐Ferreira, 2020) or changes in resource distribution (Guillermo‐Ferreira & Del‐Claro, 2011b), can be compared with model predictions to assess accuracy. Automated video tracking systems can analyse animal interactions and movement patterns under various environmental conditions, refining the model's interaction rules (Dell et al., 2014).

Empirical parameterisation using statistical fitting techniques can estimate key model parameters, such as interaction ranges, movement speeds, and attraction/repulsion strengths (Delgado et al., 2014). Sensitivity analyses can identify which parameters have the most significant impact on model outcomes, helping prioritise specific empirical data collection (Marcot et al., 2015). Cross‐validation, where empirical data is split into training and testing sets, can help calibrate and evaluate the model's predictive performance (Liu et al., 2019). Hence, an iterative process of comparing model predictions with new empirical data can lead to successive refinements, ensuring the model remains robust and accurate.

Using the model to simulate various field conditions and comparing predictions with actual field data can test the model's robustness. Field experiments designed based on model predictions can test specific scenarios, such as the effect of changing resource distribution on territorial behaviour. For example, equipping dragonflies with miniature GPS trackers (LeNaour et al., 2019) to record movements and interactions in different habitats can validate movement rules and interaction parameters. Long‐term population studies gathering data on territory sizes, mating success, and survival rates can evaluate the fitness outcomes predicted by the model under different behavioural strategies. Incorporating these empirical approaches allows the model to be continuously refined and validated, ensuring it remains relevant and accurate in describing real‐world animal behaviour and territorial dynamics.

## 4 CONCLUSIONS

A new model of territorial male competition based on the Voronoi diagram and Delaunay triangulation is proposed. It applies a simulation of animal motion utilising technique of the movable cellular automata, allowing for the evaluation of the consequences of interaction between animals with different behavioural traits and strategies. We finally suggest that this type of modelling technique may be important to tackle hard and tricky challenges when studying animal territoriality. To pinpoint theoretical and empirical data to understand the distribution, occurrence and space use of territorial animals is imperative to integrate tools that emerge as potential allies to highlight the importance of the complicated behavioural strategies animals exhibit in nature in future models.

## AUTHOR CONTRIBUTIONS

**Rhainer Guillermo‐Ferreira:** Conceptualization (equal); validation (equal); visualization (equal); writing – original draft (equal); writing – review and editing (equal). **Alexander E. Filippov:** Conceptualization (equal); data curation (equal); formal analysis (lead); investigation (lead); methodology (lead); software (equal); writing – original draft (equal); writing – review and editing (equal). **Alexander Kovalev:** Formal analysis (equal); investigation (equal); validation (equal); visualization (equal); writing – original draft (equal); writing – review and editing (equal). **Stanislav N. Gorb:** Conceptualization (equal); project administration (lead); resources (lead); supervision (lead); validation (equal); visualization (equal); writing – original draft (equal); writing – review and editing (equal).

## FUNDING INFORMATION

Funded by Conselho Nacional de Desenvolvimento Científico e Tecnológico productivity grant (312847/2022‐0).

## CONFLICT OF INTEREST STATEMENT

There are no conflicts of interest.

## Supporting information


Video S1–S4.

## Data Availability

All data is available in supplementary files.
