# Supplementary figures and images for: Voronoi diagrams and Delaunay triangulation for modelling animal territorial behaviour
Source: Ecol Evol. 2024 Jul 23;14(7):e11715. doi: 10.1002/ece3.11715 (PMC11263813; doi:10.1002/ece3.11715)

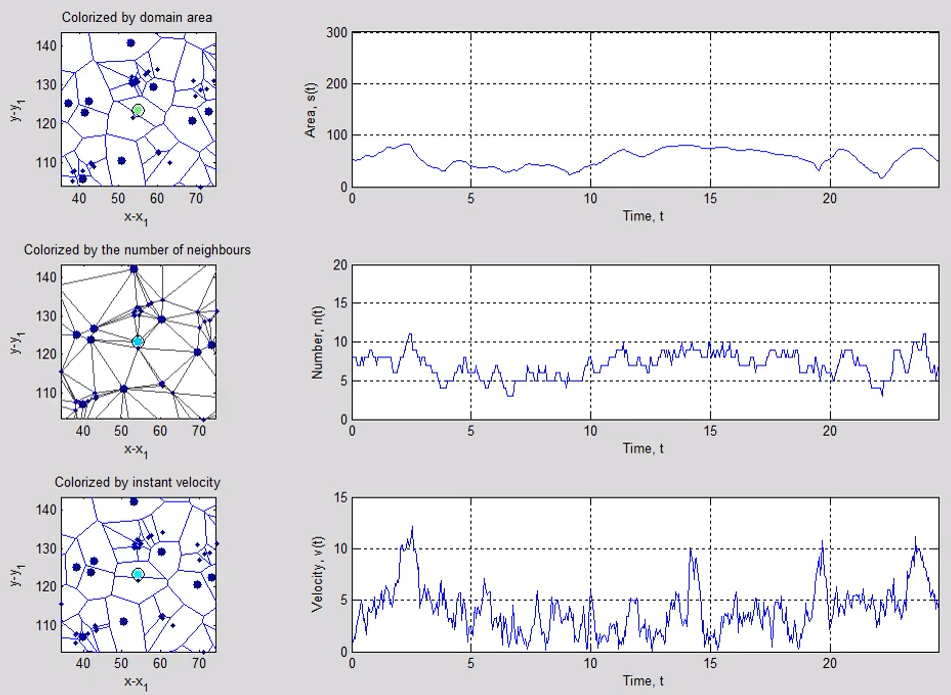

Supplement: Supplementary file 1 — Video S1–S4. [file ECE3-14-e11715-s001.zip › Fig4.PNG]

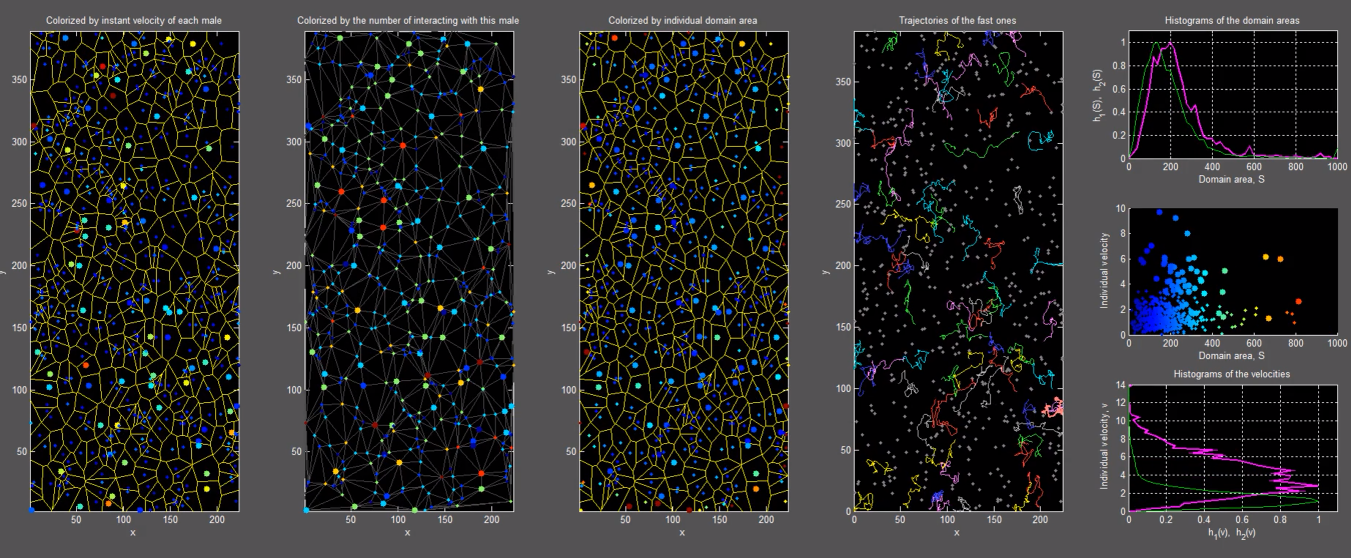

Supplement: Supplementary file 1 — Video S1–S4. [file ECE3-14-e11715-s001.zip › Fig1.PNG]

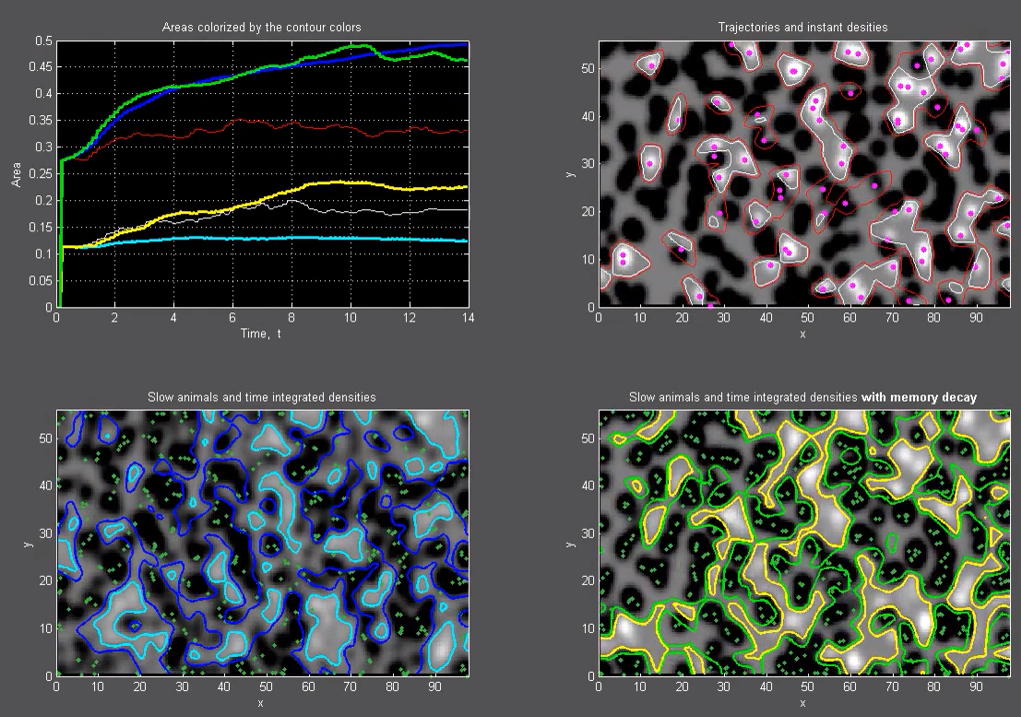

Supplement: Supplementary file 1 — Video S1–S4. [file ECE3-14-e11715-s001.zip › Fig2.PNG]

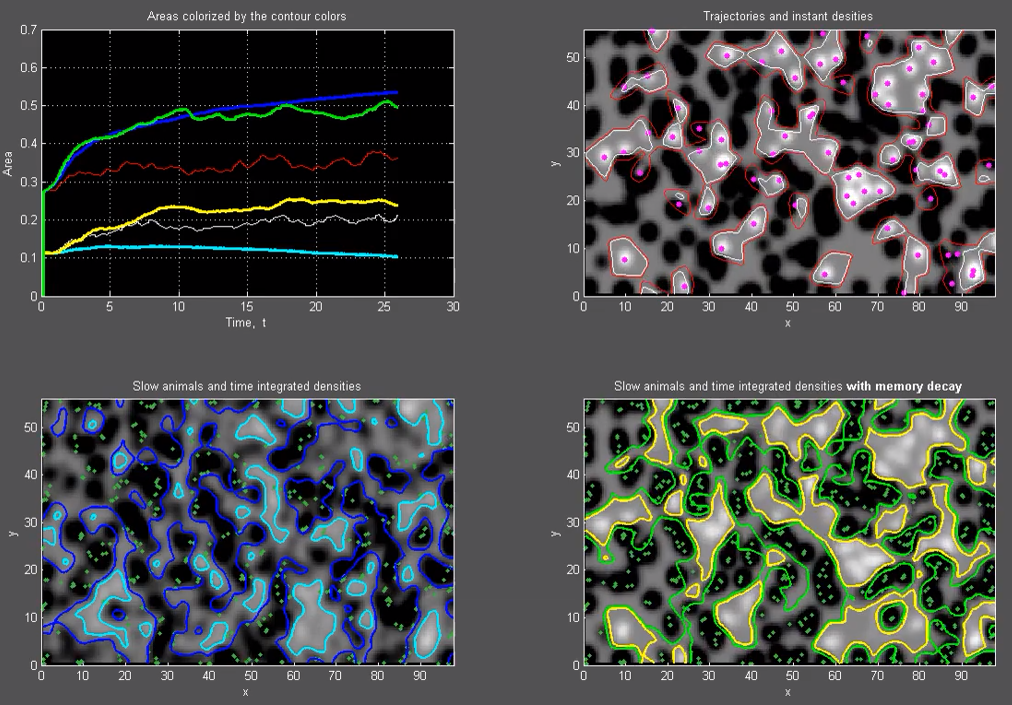

Supplement: Supplementary file 1 — Video S1–S4. [file ECE3-14-e11715-s001.zip › Fig3.PNG]
